# Genomic evaluation of carcass traits of Korean beef cattle Hanwoo using a single-step marker effect model

**DOI:** 10.1093/jas/skad104

**Published:** 2023-04-02

**Authors:** Yangmo Koo, Hatem Alkhoder, Tae-Jeong Choi, Zengting Liu, Reinhard Reents

**Affiliations:** Genetic and IT Solutions Department, Korea Animal Improvement Association, Seocho 06668, Korea; IT Solutions for Animal Production, Division of Genetic Evaluation and Biometrics, Heinrich-Schroeder-Weg 1, D-27283 Verden, Germany; Animal Genetics and Breeding Division, National Institute of Animal Science, Cheonan 31000, Korea; IT Solutions for Animal Production, Division of Genetic Evaluation and Biometrics, Heinrich-Schroeder-Weg 1, D-27283 Verden, Germany; IT Solutions for Animal Production, Division of Genetic Evaluation and Biometrics, Heinrich-Schroeder-Weg 1, D-27283 Verden, Germany

**Keywords:** beef cattle, carcass traits, genomic evaluation, single-step model

## Abstract

Hanwoo beef cattle are well known for the flavor and tenderness of their meat. Genetic improvement programs have been extremely successful over the last 40 yr. Recently, genomic selection was initiated in Hanwoo to enhance genetic progress. Routine genomic evaluation based on the single-step breeding value model was implemented in 2020 for all economically important traits. In this study, we tested a single-step marker effect model for the genomic evaluation of four carcass traits, namely, carcass weight (CW), eye muscle area, backfat thickness, and marbling score. In total, 8,023,666 animals with carcass records were jointly evaluated, including 29,965 genotyped animals. To assess the prediction stability of the single-step model, carcass data from the last 4 yr were removed in a forward validation study. The estimated genomic breeding values (GEBV) of the validation animals and other animals were compared between the truncated and full evaluations. A parallel conventional best linear unbiased prediction (BLUP) evaluation with either the full or the truncated dataset was also conducted for comparison with the single-step model. The estimates of the marker effect from the truncated evaluation were highly correlated with those from the full evaluation, ranging from 0.88 to 0.92. The regression coefficients of the estimates of the marker effect for the full and truncated evaluations were close to their expected value of 1, indicating unbiased estimates for all carcass traits. Estimates of the marker effect revealed three chromosomal regions (chromosomes 4, 6, and 14) harboring the major genes for CW in Hanwoo. For validation of cows or steers, the single-step model had a much higher *R*^*2*^ value for the linear regression model than the conventional BLUP model. Based on the regression intercept and slope of the validation, the single-step evaluation was neither inflated nor deflated. For genotyped animals, the estimated GEBV from the full and truncated evaluations were more correlated than the estimated breeding values from the two conventional BLUP evaluations. The single-step model provided a more accurate and stable evaluation over time.

## Teaser

Will the breeding of the Korean beef cattle Hanwoo be as successful as the Japanese Wagyu? The single-step model for the genomic evaluation of four carcass traits yielded a more accurate and stable evaluation over time than the conventional BLUP model.

## Introduction

Hanwoo is an indigenous beef breed in the Republic of Korea and its meat is recognized for its tenderness, flavor, and taste (Nwogwugwu et al. 2020). Beginning in the 1980s, an extensive genetic improvement program was implemented in Hanwoo cattle, using routine performance recordings, and progeny tests. Rapid genetic progress has been achieved by applying the best linear unbiased prediction (BLUP) model for the routine genetic evaluation of economically important traits (Nwogwugwu et al. 2020). To hasten genetic progress, a routine genomic evaluation system was set up for Hanwoo in 2020 by the Korean Animal Improvement Association (KAIA).

Since the invention (Meuwissen et al. 2001) and introduction (VanRaden 2008) of genomic selection and evaluation, research projects on the routine genomic evaluation of ­Hanwoo (Park et al. 2020) have been based on the single-step genomic BLUP model (ssGBLUP, Aguilar et al. 2010; Christensen and Lund 2010). Because the single-step model considers all phenotypes, genotypes, and pedigrees simultaneously, it has become the model of choice for routine genetic and genomic evaluation in diverse livestock species (Misztal et al. 2020). In this study, we chose an alternative to the single-step model that allows direct estimation of the effects of single nucleotide polymorphism (SNP): the single-step SNP BLUP model (ssSNPBLUP, Liu et al. 2014). Vandenplas et al. (2019) demonstrated the computational efficiency of the ssSNPBLUP model over other single-step models for large-scale genomic evaluations. Alkhoder et al. (2022) showed the advantages of ssSNPBLUP over the multistep SNP BLUP model for the genomic evaluation of conformation traits in the German Holstein population.

Over the past decade, numerous studies have been conducted on the genomic selection of Hanwoo (Lee et al. (2017)Mehrban et al. (2019)). These studies primarily used genotype data of bulls with progeny and a limited number of steers with their own performance data. To the best of our knowledge, no study has been published on the genomic evaluation of Hanwoo based on a large number of genotyped cows, steers, and young animals. Recently, Mehrban et al. (2021) demonstrated the enhanced accuracy of single-step genomic prediction using a multi-trait model compared to that using a single-trait model.

The objectives of this investigation were as follows: 1) To apply the ssSNPBLUP model for the multi-trait genomic ­evaluation of carcass traits in Hanwoo using genotype data from cows, steers, and young animals., 2) To conduct a genomic validation by simulating a previous genomic evaluation conducted 4 yr ago., and 3) To identify chromosomal regions important for carcass traits in Hanwoo.

## Materials and Methods

### Data and materials

Our genomic evaluation of the Hanwoo animals followed the publicly available animal care and use standards in Korea and Germany, equivalent to the institutional animal care and use committee (IACUC) in the United States. Four carcass traits were routinely collected from steers and cows of Hanwoo cattle: carcass weight (CW), eye muscle area (EMA), backfat thickness (BF), and marbling score (MS). These carcass traits are among the economically important traits in Hanwoo (Kim et al. 2021; Bedhane et al. 2019; Mehrban et al. 2019; Nwogwugwu et al. 2020; Son et al. 2020; Seo et al. 2022). An updated scheme for data recording were introduced for Hanwoo in 2008. In recent years, the Korean breeding organization KAIA has begun genotyping male and female animals for genomic selection. Table 1 shows the number of steers and cows with recorded data on all four carcass traits, and the number of genotyped animals by year of birth. In total, carcass trait data were available for 8,023,666 cows and steers.

**Table 1.** Numbers of steers and cows with recorded carcass traits in Hanwoo beef cattle

Year of birth	Cows with phenotype		Steers with phenotype		Genotyped	
	All	Genotyped	All	Genotyped	Males	Females
≤2000	178,798		15,249	1	4	29
2001	38,539		4,509	0	6	1
2002	56,219	4	9,827	3	4	5
2003	81,408	4	15,473	2	2	7
2004	116,445	1	16,741	17	17	9
2005	167,182	5	37,072	7	7	13
2006	215,651	22	78,761	13	13	42
2007	246,629	33	139,766	6	7	86
2008	278,418	65	177,218	15	16	136
2009	287,913	100	189,280	18	18	265
2010	329,710	229	236,620	24	25	562
2011	348,889	358	258,187	31	37	758
2012	364,775	513	282,188	40	41	1,253
2013	312,830	360	268,476	58	58	971
2014	297,521	221	280,902	873	876	640
2015	282,585	230	284,860	2,822	2,835	1,022
2016	261,959	114	302,348	905	932	1,542
2017	221,223	203	319,816	393	409	2,577
2018	158,037	158	341,153	3	17	3,287
2019	98,103	103	358,429	1	3	3,755
2020	15,523	21	47,529	22	785	4,398
2021	450		455		27	2,389
2022					1	78
Total	4,358,807	2,744	3,664,859	5,254	6,140	23,825

Over the last 10 yr, the number of genotyped female animals has increased continuously. The total number of genotyped animals was 29,965 using three SNP chips: Affymetrix Axiom Bovine 60 K chip with 65,003 SNPs, Illumina Bovine 50 K chip version 1, and a customized Hanwoo 50 K chip. Approximately 85%, 8%, and 7% of all animals were genotyped using Affymetrix Axiom Bovine 60 K, Illumina Bovine 50 K, and Hanwoo 50 K chips, respectively. A total of 47,200 SNP markers selected for genomic evaluation were present on the major Affymetrix Axiom Bovine 60 K chip. The Hanwoo 50 K and Illumina 50 K chips contained 41,158 and 35,473 SNP, respectively, in common with the selected final SNP set. Missing SNP genotypes were imputed using the findhap software (VanRaden et al. 2013).

Ancestors of the genotyped or phenotyped animals presented in Table 1 were traced back to the pedigree as far as possible. The total number of animals included in the final pedigree of the single-step evaluation was 13,534,295. Thirty-three phantom parent groups were defined for the missing parents of animals based on the four selection paths and birth years of the animals.

### Multi-trait single-step SNP BLUP model

For an animal with phenotypic records of carcass traits, the following multi-trait model was used, as described by Son et al. (2020):


$$\begin{array}{l} y=X_{f}f+X_{b}b+u+e \end{array}$$ [1]

where **y** is a vector of observations of the four carcass traits of the animal, **f** is a vector of fixed effects of herd-year and year-season on slaughter and sex of the animal, **b** is a vector of linear regression on the age of the animal at slaughter, **u** is a vector of breeding values of the four traits of the animal, and **e** is a vector of random error effects associated with the observations. **X**_*f*_ is the incidence matrix for the classification fixed effects *f*, **X**_*b*_ is the design matrix for the regression effect *b*.

If an animal is genotyped, the genomic breeding value (GEBV) of a carcass trait *i* can be decomposed into two components according to the ssSNPBLUP model (Liu et al. 2014). Therefore, model 1 can also be expressed for genotyped animals as follows:


$$\begin{array}{l} y=X_{f}f+X_{b}b+Zg+a+e\; \end{array}$$ [2]

where **g** is a vector of all SNP marker effects, **a** is a vector of the residual polygenic effects (RPG; Liu et al. 2011) of the genotyped animals, and **Z** is the design matrix containing SNP genotype data of the animals. It was assumed that RPG effects explained 20% of the additive genetic variance, *k* = 0.2. This RPG parameter suggests that the SNP marker genotype information accounted for 80% of the additive genetic variance. Based on our validation study, the parameter *k* = 0.2 seemed to be an optimal value for the evaluated traits of Hanwoo. Let $\sigma_{u_{i}}^{2}$ represent the total additive genetic variance of the *i*-th carcass trait; then, the average genetic variance of SNP markers was calculated using the ssSNPBLUP model as follows:


$$\begin{array}{l} \sigma_{SNP}^{2}=(1-k)\sigma_{u_{i}}^{2}/(\underset{j=1}{\overset{m}{\sum }}\;2p_{j}(1-p_{j})) \end{array}$$ [3]

where *m* = 47,200 is the number of SNP markers, and $p_{j}$ is the allele frequency of the *j*th SNP marker.

The variance and covariance components for additive genetic and random error effects were obtained from a recent study (Son et al. 2020) on carcass traits of Hanwoo for single-step evaluation. The heritability values ranged from 0.28 to 0.48. Moderate positive genetic correlations were found between all trait pairs except for low negative correlations between BF and EMA or MS.

The software package MiX99 (Strandén and Lidauer, 1999) was used for the single-step evaluation. The ssSNPBLUP model was implemented in MiX99 in a special manner with SNP markers treated as if they were animals with neither parents nor progeny known (Alkhoder et al. 2022).

### Genomic validation of the single-step SNP BLUP model

To assess the stability of genomic prediction in Hanwoo, genomic validation was performed by simulating a previous genomic evaluation conducted 4 yr ago. All phenotypic records of carcass traits were removed for cows or steers born after 2016 (Table 1). Genotyped cows and steers with their own carcass traits, born in 2017 and thereafter, were chosen as validation animals. There were 480 and 419 validation cows and steers, respectively. Owing to the lack of genotype data, the total number of genotyped bulls with progeny was 355 in the full dataset. The number of genotyped bulls, defined as validation bulls, was 83. Therefore, we decided not to use bulls as validation animals for genomic validation.

To compare the predictive ability of the single-step model 1, we conducted a conventional BLUP evaluation using the same animals as those used for the single-step evaluation, with young animals included in the full and truncated datasets.

## Results and Discussion

The pedigree error rate in Hanwoo was investigated using a genotyped subpopulation. Among the 29,965 genotyped animals, 25,501 and 6,604 were sire- and dam-genotyped, respectively. After parentage verification and discovery in the genotyped subpopulation, we obtained a pedigree error rate of 6.9% on the sire side and 6.1% on the dam side. Pedigree error rates in the genotyped population of Hanwoo seem to be within the range reported for beef or dairy cattle in literature.

All single-step and conventional BLUP evaluations with the full and truncated datasets were run on a Linux computer with 128 GB random access memory and 12 cores. For the single-step evaluation using the full dataset, the total number of effects to be estimated was 57,260,624. The peak memory usage was approximately 30.8 Gb and the total clock time was approximately 1 h to reach a predefined convergence criterion after 549 iterations. With the same dataset, the ssGBLUP model required a similar clock time to reach the same predefined convergence criterion, but with a smaller peak memory usage of 10.3 Gb. The ssSNPBLUP model required more memory than the ssGBLUP model, because the number of SNP markers was greater than the number of genotyped animals. With more genotyped animals added later, the ssSNPBLUP model will be more efficient in terms of memory usage (Alkhoder et al. 2022) compared to the ssGBLUP model.

Four evaluations were conducted: single-step evaluations with the full and truncated datasets, and conventional BLUP evaluations with the full and truncated datasets. The ­solutions of the mixed model equations for additive genetic effects were adjusted for the four evaluations for a common base population of animals (born in 2015) with carcass records. For a direct comparison of the evaluation results across the four traits, the adjusted GEBV were divided by the respective genetic standard deviation of each trait.

### Estimation of SNP effects

**Figure 1.**
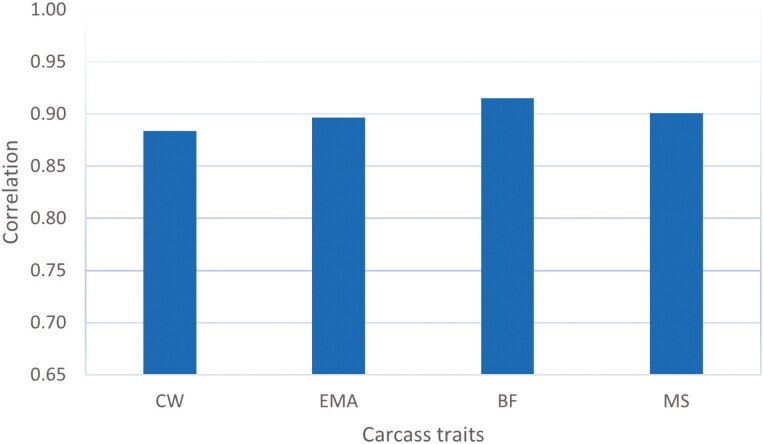
Correlation of estimates of the marker effect of the single-step model between the full and truncated evaluation for the carcass traits in Hanwoo.

**Figure 2.**
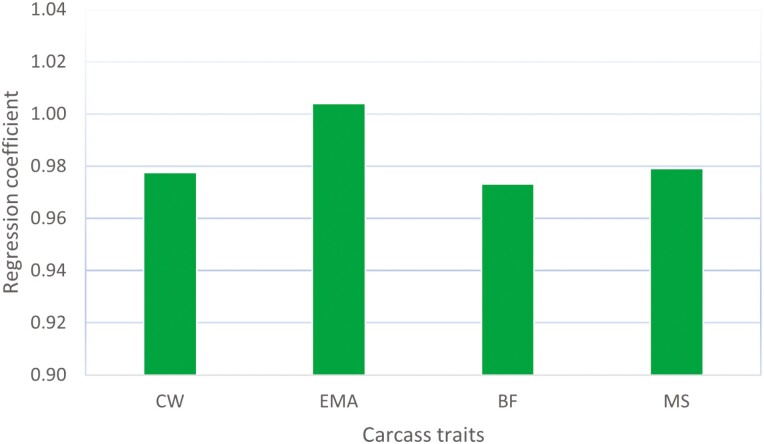
Regression of estimates of the marker effect of the single-step full evaluation on the truncated evaluation for carcass traits in Hanwoo.

The single-step model ssSNPBLUP (Liu et al. 2014) directly estimated the effects of all the SNP markers, besides the GEBV of all the animals in the pedigree and all the fixed effects of model 1. We compared the estimates of SNP effects between the full and truncated evaluations of the single-step model. The stability of the GEBV over time is one of the most important quality control criteria for routine genomic evaluations. If the estimates of SNP effects are highly correlated between two evaluations at earlier and later times, the GEBV of the animals will also be highly correlated, particularly for young animals without phenotypic data. A high SNP-effect correlation between the two evaluations at different times is desirable for stable genomic prediction. Figure 1 shows the correlation coefficients of estimates of SNP effects between the full and truncated evaluations for each trait. The correlation coefficients of the SNP effects range from 0.88 to 0.92 for traits CW and BF, respectively. The regression coefficients of the estimates of SNP effects of the full and truncated evaluations are shown in Figure 2. The regression coefficients are close to their expected values of 1, varying from 0.97 for BF and 1.00 for EMA. The regression slopes indicate that the estimates of SNP effect were neither inflated nor underestimated. The relatively high correlations of estimates of the SNP effects in Figure 1 may be, to a certain degree, caused by the high proportion of common phenotypic data shared between the truncated and full evaluations. However, the high percentage of common phenotypic data between the full and truncated evaluations should have a negligible impact on the regression coefficients of the estimates of SNP effects, as shown in Figure 2.

**Figure 3.**
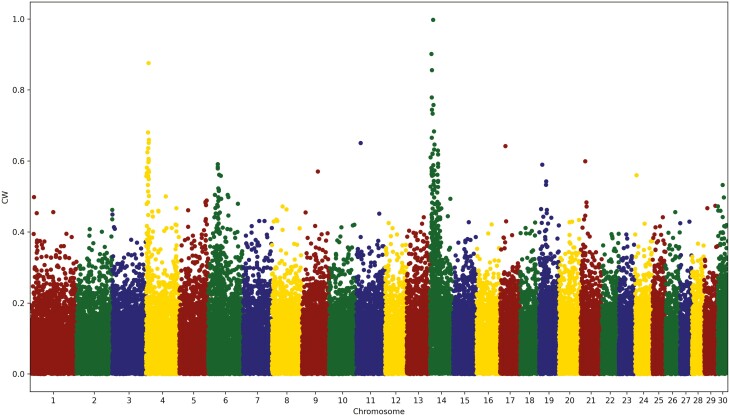
Plot of estimates of the marker effect, expressed in the unit of marker genetic standard deviation, for carcass weight in Hanwoo.

**Figure 4.**
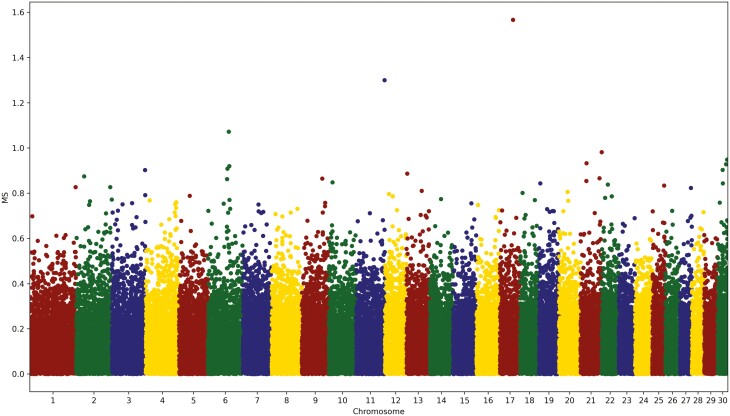
Plot of estimates of the marker effect, expressed in the unit of marker genetic standard deviation, for marbling score in Hanwoo.

In contrast to the usual single-marker genome-wide association study (GWAS), the genomic model [1] jointly estimated the effects of all 47,200 SNP markers. Figures 3 and 4 show the plots of SNP effects for the CW and MS traits, respectively. All the estimates of the SNP effects were divided by the genetic standard deviation of SNP markers, $\sigma_{SNP}^{}$, such that they can be compared directly across the four traits. In contrast to the regular GWAS, we did not include or exclude the SNP markers by explicitly calculating the probability of the *t*-test for the effect estimate of every SNP marker because the focus of our genomic prediction was not on the significance testing of the SNP markers and both significant and nonsignificant SNP were included. Aguilar et al. (2019) provided a statistical method for approximating the *P* values of the estimates of SNP effects from the ssGBLUP model. Further research is necessary to develop similar methods for approximating the *P* values or prediction error variances of the estimates of SNP effects achieved using our ssSNPBLUP model. As shown by Liu et al. (2011), the variance in the estimates of SNP effects increased with the size of the reference population. More SNP markers with larger effects would yield an even larger estimate of effect with an increasing number of reference animals. Our plots of SNP effects resembled those of regular Manhattan plots because the standard errors of the estimates of SNP effects of all the SNP markers did not differ significantly. Our plots of SNP effects provide sufficient information to detect chromosomal regions with large effects. Chromosomes 4, 6, and 14 appear to harbor large genes or mutations in the CW trait (Figure 3). Compared to CW, the SNP effects of trait MS have a higher variation, with many SNP effects greater than 50% of $\sigma_{SNP}^{}$.

In their GWAS, Lee et al. (2013) also reported SNP markers with large effects on chromosome 14 in CW. Similar to our plot for CW, Kim et al. (2021) found peaks on chromosomes 4 and 6, in addition to the highest peak on chromosome 14. These two previous studies confirmed that our chromosomal regions harbored major genes or mutations in CW.

In contrast to CW, no dominant peak was observed in the plot for trait MS (Figure 4); however, many SNP markers with medium-sized effects were identified for trait MS. Similar SNP effects were also revealed in studies by Bedhane et al. (2019) and Kim et al. (2021).

For trait BF (data not shown), we also identified a region on chromosome 19 as described by Kim et al. (2021). Our plots of SNP effects for MS resembled that of Kim et al. (2021). As shown in Figure 4, for the MS trait, no major loci appeared to exist for the EMA trait (data not shown). However, there were many SNP markers with medium-sized effects, with a standardized marker effect between 0.3 and 0.8. We did not see a strong indication of major genes for trait EMA in our study, which does not agree with the findings of Seo et al. (2022), who identified significant regions on chromosomes 3 and 14 via a single-marker GWAS. One reason for the difference in significant chromosome regions for trait EMA may be that most genotyped animals were cows in our study versus bulls by Seo et al. (2022). Additional research with more genotyped animals is necessary to verify our results concerning the estimates of SNP effects for trait EMA in Hanwoo.

### Genomic validation results

A linear regression test (LR test, Legarra and Reverter, 2018) was performed between the GEBV of the single-step full and truncated evaluations of validation cows or steers. For comparison with the single-step model, the LR test was applied to the conventional BLUP EBV with the full and truncated datasets. Table 2 shows the results of genomic validation for the two groups of validation animals of Hanwoo for carcass traits.

**Table 2.** Results of the linear regression test of the carcass traits in Hanwoo for validation cows or steers

Trait	Model	Regression intercept b_0_	Regression slope b_1_	*R* ^*2*^ value
*480 validation cows*				
CW	SSM	−0.10	0.97	0.74
	CONV	−0.09	0.94	0.53
EMA	SSM	−0.04	1.02	0.74
	CONV	−0.05	1.02	0.56
BF	SSM	−0.05	1.01	0.69
	CONV	−0.07	1.02	0.51
MS	SSM	−0.03	1.00	0.63
	CONV	−0.08	1.01	0.40
*419 validation steers*				
CW	SSM	−0.03	0.98	0.75
	CONV	−0.04	0.98	0.59
EMA	SSM	0.10	1.03	0.70
	CONV	0.09	1.01	0.53
BF	SSM	−0.05	1.03	0.71
	CONV	−0.04	1.06	0.58
MS	SSM	0.05	1.06	0.63
	CONV	0.05	1.06	0.40

SM and CONV stand for single-step and conventional best linear unbiased prediction model. Regression intercept b_0_ is expressed in the unit of genetic standard deviation.

It can be seen that the *R*^2^ values of the linear regression model, being related to the accuracy of genomic prediction, are higher for the single-step than for the conventional BLUP model in either group of validation animals for each of the four carcass traits. The difference in *R*^2^ values between the two models ranges from 0.13 for trait BF of validation steers to 0.23 for trait MS of validation cows. The single-step model had a markedly higher correlation of GEBV compared to the conventional BLUP model, suggesting a higher stability of GEBV in the single-step evaluation over time.

The regression intercept in Table 2, b_0_, is expressed in the unit of the genetic standard deviation of the corresponding trait. The lowest b_0_ value, −0.03, is found for trait MS and CW of validation cows and steers, respectively, whereas the highest value was 0.10 for trait EMA of validation steers. A slight overestimation of the intercept, shown as a negative value, was observed in all combinations of traits and animal groups, except for validation steers in EMA and MS, which showed a slight underestimation with a positive b_0_ value. Overall, the regression intercept values do not deviate significantly from the expected value of 0.

The regression slope, *b*_1_, indicates the inflation of genomic prediction if *b*_1_ < 1, and an underestimation of genomic prediction if *b*_1_ > 1. None of the *b*_1_ values in Table 2 deviated markedly from the expected value of 1, suggesting that the single-step genomic prediction or conventional BLUP prediction was neither inflated nor deflated. Relative to the single-step evaluation, conventional BLUP evaluation showed a greater deviation in *b*_1_ from the expected value of 1.

### Genetic trends in steers

**Figure 5.**
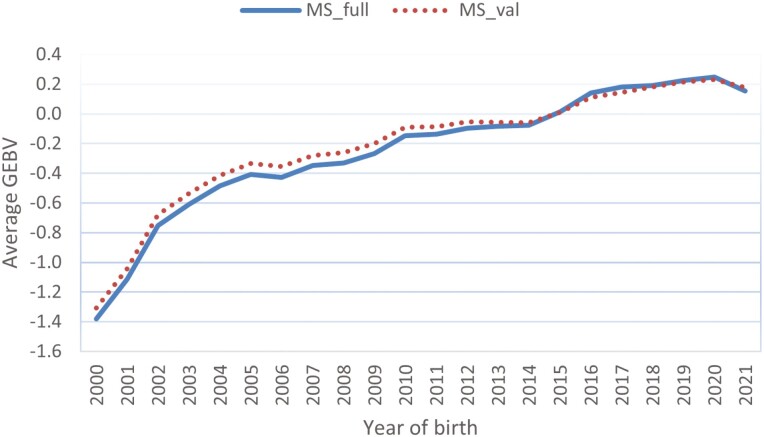
Genetic trend in steers with marbling score records from the single-step genomic evaluations with the full and truncated datasets. MS_full and MS_val represent genomic evaluations using the full and truncated datasets, respectively. The estimated GEBV are expressed as genetic standard deviations.

The average GEBV of all steers with their own carcass records, genotyped, and non-genotyped, was calculated by the year of birth for the single-step evaluations with full and truncated datasets. Figure 5 shows the genetic trends in steers of the single-step model for trait MS. Notable genetic progress has been made in trait MS of Hanwoo steers, amounting to 1.6 genetic standard deviations from birth year 2000 to 2020. The single-step evaluation with the truncated dataset exhibited a slightly lower trend than that of the full evaluation. The genetic trends of the conventional BLUP evaluation (data not shown here) seem to be highly similar to those of the single-step model, suggesting that genomic selection started only recently in Hanwoo, with no clear impact.

**Figure 6.**
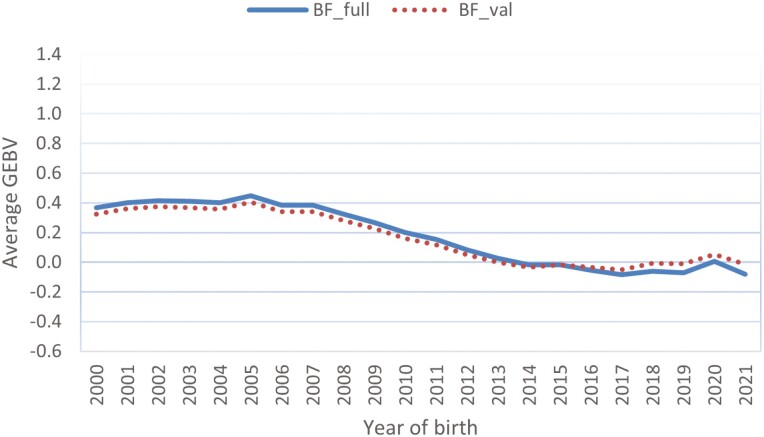
Genetic trend in steers with backfat thickness records from the single-step genomic evaluations with the full and truncated datasets. BF_full and BF_val represent genomic evaluations using the full and truncated datasets, respectively. The estimated GEBV are expressed as genetic standard deviations.

Figure 6 shows the genetic trends of BF in Hanwoo steers. The decreasing trend in trait BF indicates favorable genetic progress since the update of the data-recording scheme in 2008. A flat genetic trend was observed before the birth year 2007/2008. We also observed a nearly flat genetic trend curve in the recent birth years from 2016 and later, reflecting less emphasis on the selection objective imposed on trait BF. As shown in Figure 5 for trait MS, we observed a slightly lower genetic trend for the single-step evaluation with the truncated dataset than with the full dataset. In comparison to trait MS, less genetic progress has been made in trait BF, which can be explained by the lower heritability value of BF and lower weight of BF in the carcass index of Hanwoo.

Similar genetic trends were found in steers for traits CW, EMA, and MS (data not shown). For the CW trait, a larger trend was observed in the single-step evaluation, notably with the truncated dataset, than with the full dataset. Compared to steers with carcass data, cows with their own carcass records seem to have less genetic progress in trait MS but similar progress in traits BF, EMA, and CW.

### Correlations of predictions between the full and truncated evaluations

**Figure 7.**
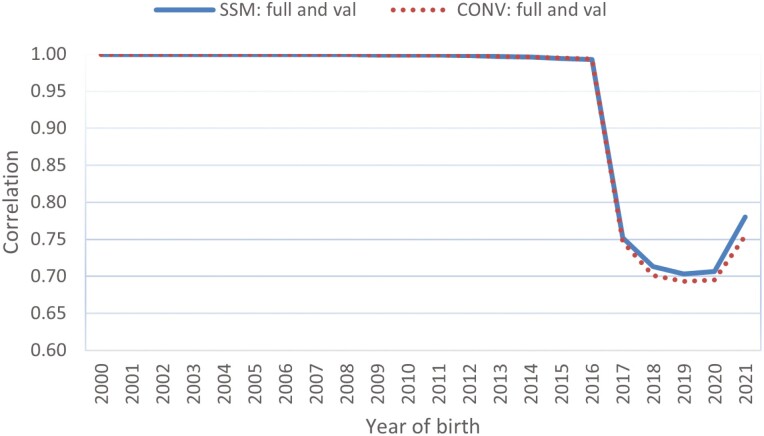
Correlation of estimated genomic or conventional best linear unbiased prediction (BLUP) breeding values of steers with carcass weight records between the full and truncated datasets for the single-step or conventional BLUP model. SSM: full and val or CONV: full and val represent the correlations between the full and truncated evaluations for the single-step model and the conventional BLUP model, respectively.

The GEBV correlation between the full and truncated evaluations of data from 4 yr ago indicates the predictive ability of the single-step genomic model. Figure 7 shows the correlations of the GEBV or conventional BLUP EBV of steers with the CW records between the truncated and full evaluations for the single-step model (solid blue line) or the conventional BLUP model (dotted red line). Steers born in 2016 or before have CW records in both the truncated and full datasets, and the correlations of GEBV or EBV between the two evaluations are unity for either model. Steers born in 2017 and later had CW records in the full evaluation; however, no data in the truncated evaluation showed a much lower GEBV or EBV correlation, as low as 0.70. Despite a smaller difference in the correlations, the GEBV of the truncated evaluation were consistently higher and more correlated with the full evaluation for the single-step model than those for the conventional BLUP model. The higher GEBV correlation indicated a higher prediction accuracy of the single-step model than that of the conventional BLUP model, despite the relatively small difference in the correlations owing to the limited number of genotyped animals with CW records. Similar correlations were observed for the other three traits (BF, EMA, and MS) for steers with carcass records.

**Figure 8.**
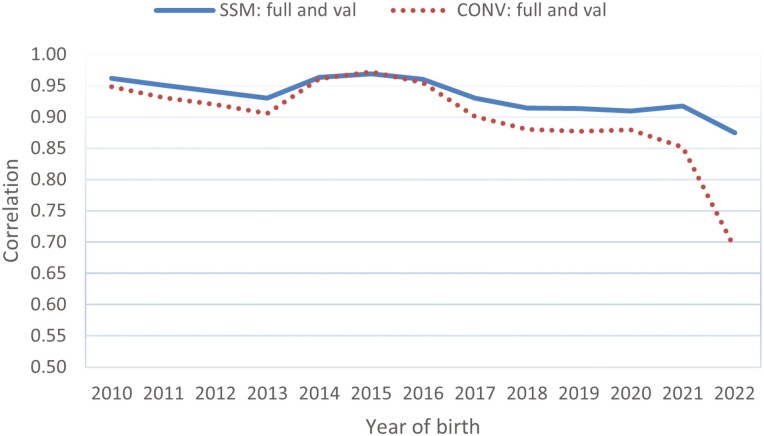
Correlation of estimated genomic or conventional best linear unbiased prediction (BLUP) breeding values of genotyped animals for carcass weight between the full and truncated datasets for the single-step or conventional BLUP model. SSM: full and val or CONV: full and val represent the correlations between the full and truncated evaluations for the single-step model and the conventional BLUP model, respectively.

As shown in Table 1, some of the 29,965 genotyped animals, including those born in the early years, had no carcass records. Figure 8 shows the GEBV and EBV correlations between the full and truncated evaluations for the single-step model (solid blue line) and conventional BLUP model (dotted red line), respectively. Across all birth years of the genotyped animals, the single-step model showed a markedly higher GEBV correlation between the two evaluations than the conventional BLUP model. Only in the birth years 2014 through 2016 did the two models give equal GEBV or EBV correlations between the two datasets because these three birth years have the most genotyped animals with their own carcass records in either the full or the truncated evaluation. The higher correlations between the full and truncated evaluations indicated a higher accuracy of genomic prediction and a more stable genomic prediction over time for the single-step model.

## Abbreviations

BF: backfat thickness
BLUP: best linear unbiased prediction
CW: carcass weight
EBV: estimated breeding values
EMA: eye muscle area
GEBV: genomic estimated breeding values
GWAS: genome-wide association study
KAIA: Korea Animal Improvement Association
LR: linear regression
MS: marbling score
RPG: residual polygenic effect;
ssGBLUP: single-step genomic best linear unbiased prediction
ssSNPBLUP: single-step single nucleotide polymorphism best linear unbiased prediction
SNP: single nucleotide polymorphism
